# Comparison of the Prevalence of Metabolic Disease Between Two Types of Urbanization in China

**DOI:** 10.3389/fendo.2018.00665

**Published:** 2018-11-12

**Authors:** Bing Han, Yi Chen, Jing Cheng, Qin Li, Chunfang Zhu, Yingchao Chen, Fangzhen Xia, Ningjian Wang, Yingli Lu

**Affiliations:** Institute and Department of Endocrinology and Metabolism, Shanghai Ninth People's Hospital Affiliated With Shanghai Jiaotong University School of Medicine, Shanghai, China

**Keywords:** urbanization, occupation, physical activity, blood glucose, BMI, NAFLD

## Abstract

**Objective:** China is experiencing the world's largest urbanization. There are two primary types of urbanization in China: rural-to-urban migration and *in situ* urbanization, represented by Zhejiang Shangyu (SY) and Jiangsu Nanjing (NJ), respectively. Our aim is to compare changes in the prevalence of metabolic disease between these two types of urbanization in China.

**Methods:** This is a cross-sectional study derived from the SPECT-China 2014 study. This study includes subjects and metabolic parameters from SY and NJ. Furthermore, biochemical and anthropometric indexes were taken into consideration and compared between the areas of interest.

**Results:** The prevalence rates of diabetes, prediabetes and healthy subjects were 6.5, 17.9, and 75.7% in SY and 16.0, 31.0, and 53.0% in NJ, respectively. Industrial and agricultural jobs accounted for 77.9% and 32.0% of employment in SY and NJ, respectively. Fasting plasma glucose (FPG) was higher in SY than in NJ; however, HbA1c was lower in SY than in NJ. There was a significant difference in nonalcoholic fatty liver disease (NAFLD) and healthy subjects between SY and NJ (*P* < 0.05). Significant differences were also found with respect to body mass index (BMI), waist circumference (WC) and hip circumference (HC) between these two locations (*P* < 0.001). Logistic regression analysis revealed that the prevalence of prediabetes, diabetes, overweight, obesity and dyslipidemia was higher in NJ than in SY.

**Conclusions:** *In situ* urbanization has notably changed occupational distribution. The prevalence rates of diabetes, obesity, and NAFLD were increased in rapidly urbanized areas. Thus, more attention should be paid to rapidly urbanizing areas to reduce the prevalence of metabolic disease.

## Introduction

The prevalence of non-communicable diseases (NCDs) has increased rapidly worldwide (1). In 2005, NCDs accounted for nearly 80% of deaths and 70% of total disability-adjusted life-years lost in China (1). Therefore, NCDs have become an important public health problem (2, 3). However, China is now experiencing the largest scale of accelerated urbanization in the world (4). The percentage of the urban population rose from 18% in 1978 to 56% in 2015 (4), and it is anticipated that 1 billion people will be living in urban areas by 2030 (5). The people living in urban areas will benefit from improved health services; nonetheless, these people will also have unhealthy lifestyles (6).

In China, urbanization is achieved in two ways: migration from rural to urban areas and *in situ* urbanization of rural areas (7, 8). In the latter, with the expansion of a city, rural areas become urban regions. People living in rural areas will experience considerable changes to their lifestyle (8), as urbanization continues in China and *in situ* urbanization becomes more popular. However, the influence of *in situ* urbanization on metabolism has not been thoroughly elucidated to date. Furthermore, the effect of different speeds of urbanization on metabolism should also be studied.

In the SPECT-China study, we studied the metabolic changes in subjects of eastern China. In this study, we used the survey data of two areas that were countryside environments 10 years ago and intended to explore the influence of different means of urbanization on metabolic diseases by comparing the changes in metabolic indexes in these two places. This study will help us better understand the influence of rapid urbanization on metabolism.

## Subjects and methods

### Study population

This report describes a substudy of SPECT-China (registered number: ChiCTR-ECS-14005052, www.chictr.org), representing a population-based cross-sectional survey on the prevalence of metabolic diseases and risk factors in eastern China. For this study, 1,569 subjects in Zhejiang Shangyu (SY) and 1,427 subjects in Jangsu Nanjing (NJ) were recruited. Adults who had lived in their current residence for 6 or more months were selected and enrolled in our study. Exclusion criteria included the following: excessive drinking (*n* = 159), self-reported viral hepatitis (*n* = 30) and use of corticosteroids (*n* = 10). Finally, the current study was based on a total of 2,797 subjects (1,562 in SY and 1,235 in NJ).

Zhejiang Shangyu (SY) is a county-level city. The selected three natural villages were gradually developed. In this situation, urbanization was gradually achieved by urban expansion and migration from rural to urban areas. Before 2007, the Jiangsu Nanjing (NJ) Xianhemeng community was composed of three natural villages. In 2007, the people who lived in these three villages were voluntarily relocated to form a large community. Villagers lost their land and became urban residents, and urbanization was achieved by *in situ* urbanization. In 2005, these two districts were countryside and had similar average annual incomes (7704 RMB in SY and 6225 RMB in NJ), but during the past 10 years, they experienced different development speeds. NJ rapidly changed to an urban area by *in situ* urbanization, whereas SY developed relatively slowly.

This study protocol was approved by the Ethics Committee of Shanghai Ninth People's Hospital. Written informed consent was obtained from all participants.

### Assessment of indexes

Blood sample collection and biochemical indexes were described previously (9). Liver ultrasound was performed by a sonographer (SonoSite, M-Turbo). Blood pressure and heart rate were measured by sphygmomanometer (TERUMO-Elemano) three times after resting for 5 min. The mean value of three measurements was used in the analysis. Demographic information and lifestyle risk factors were collected by trained staff using standard questionnaires. Waist circumference (WC) was measured to the nearest 0.5 cm midway between the lower edge of the last rib and the iliac crest in standing position. Wearing lightweight clothes and no shoes, height, and weight were measured to the nearest 0.5 cm and 0.1 kg, respectively.

### Definition and classification

Diabetes was defined as self-reported diabetic history or HbA1c 6.5% or greater. Prediabetes was defined as HbA1c between 5.7 and 6.4%. NGT was defined as HbA1c < 5.7% (10). According to ultrasonographic examination, fat accumulation was divided into normal, steatosis, and fatty liver (11). Body mass index (BMI) was calculated as weight (kg)/height squared (m^2^). BMI ≥ 28 kg/m^2^, 24 kg/m^2^ ≤ BMI < 28 kg/m^2^, and BMI < 24 kg/m^2^ were defined as obesity, overweight and normal, respectively. The homeostasis model assessment of insulin resistance (HOMA-IR) was calculated as fasting serum insulin (mIU/L) × FPG (mmol/L)/22.5. Occupation was defined as follows 1, farmer; 2, worker; 3, soldier; 4, cadres; 5, teacher or doctor; 6, self-employed; 7, service staff; 8, homemaker; 9, retired; 10, unemployed; 11, student; and 12, other. Finally, these occupations were summarized as industry (including 2), agriculture (including 1), service industry (including 5, 6, and 7), unemployed (including 8, 9, and 10), and other (including 3, 4, 11, 12).

### Statistical analysis

Statistical analysis was performed using IBM SPSS Statistics, Version 22 (IBM Corporation, Armonk, New York). Continuous variables were expressed as the mean ± SD. Categorical variables were expressed as a percentage (%). The Mann-Whitney *U* test and independent sample *t*-test were used for continuous data with skewed distribution and normal distribution between SY, and NJ. The Pearson chi-square test was used for categorical variables. We used linear regression analyses to investigate the association between metabolic indexes (continuous variable) and the urbanization level. Model 1 was adjusted for age and sex. Model 2 was further adjusted for HbA1c, BMI, systolic blood pressure (SBP), high density lipoprotein (HDL), low density lipoprotein (LDL), and triglyceride (TG). Because TG exhibited a skewed distribution, it was log transformed. Logistic regression analysis was also performed to investigate the association of metabolic diseases (dichotomous variable) with the urbanization level. Prediabetes, diabetes, overweight, obesity, and dyslipidemia were dependent variables. The model was adjusted for age, sex, diabetes, weight status, hypertension, and dyslipidemia. All analyses were two-sided, and *P* < 0.05 was considered significant.

## Results

### Comparison between different locations

In this study, 1,562 subjects, including 690 males (age 54.16 ± 13.41 years) and 872 females (age 53.53 ± 13.57 years), in SY and 1,235 subjects, including 302 males (age 56.47 ± 12.84 years), and 933 females (age 55.16 ± 12.42 years), in NJ were recruited. The prevalence rates of diabetes, prediabetes, and normal people in two locations were 6.5, 17.9, and 75.7% (SY) and 16.0, 31.0, and 53.0% (NJ), respectively. There was a significant difference between SY and NJ in SBP (130.03 ± 20.58 vs. 133.26 ± 20.80 mmHg, *P* < 0.001) and DBP (77.70 ± 12.23 vs. 79.67 ± 12.71 mmHg, *P* < 0.001) (Supplement Table 1).

### Alteration in the occupational composition

The different levels of urbanization also influenced the occupational composition. Industry and agriculture accounted for 77.9% of SY residents but only 32.0% of NJ residents (*P* < 0.001). The proportions of individuals in the service industry were 6.5% and 18.0% in SY and NJ (*P* = 0.01), respectively. Moreover, the unemployment rates were 12.1% and 37.8% in SY and NJ (*P* < 0.001), respectively, (Supplement Figure 1).

### Comparison of glucose metabolism

Overall, FPG was higher in SY than in NJ (5.82 ± 1.03 vs. 5.54 ± 1.65, *P* < 0.001), except in subjects older than 70 years. This trend was more apparent in females than in males. In contrast, HbA1c was lower in SY than in NJ (5.37 ± 0.80 vs. 5.83 ± 1.05, *P* < 0.001) for all age and sex subgroups. Moreover, there was no significant difference in HOMAIR between SY and NJ (Table 1). According to glucose status, subjects were divided into diabetic, prediabetic and normal groups. Among diabetics, although the average values of HbA1c and FPG in NJ were higher than those of SY, there was still no significant difference. Among prediabetics, only FPG exhibited a significant difference between SY and NJ. However, among normal subjects, HbA1c in SY was lower than in NJ, whereas FPG in SY was higher than that in NJ (Supplement Table 2).

**Table 1:** Comparison of glucose metabolism indexes in these two places.

	**FPG**			**HbA1c**			**HOMAIR**		
	**SY**	**NJ**	***P***	**SY**	**NJ**	***P***	**SY**	**NJ**	***P***
Overall	5.82 ± 1.03	5.54 ± 1.65	<0.001	5.37 ± 0.80	5.83 ± 1.05	<0.001	1.60 ± 1.64	1.60 ± 1.26	0.918
**AGE (YEARS)**									
<40	5.35 ± 0.67	4.84 ± 0.43	<0.001	4.97 ± 0.55	5.19 ± 0.38	<0.001	1.64 ± 1.13	1.32 ± 0.78	0.004
40–50	5.61 ± 0.88	5.22 ± 1.33	<0.001	5.20 ± 0.67	5.51 ± 0.75	<0.001	1.60 ± 1.53	1.38 ± 0.90	0.059
50–60	5.93 ± 1.13	5.60 ± 1.75	0.002	5.52 ± 0.88	5.92 ± 1.13	<0.001	1.66 ± 2.17	1.51 ± 0.91	0.284
60–70	6.01 ± 0.98	5.76 ± 1.67	0.010	5.49 ± 0.78	6.07 ± 1.09	<0.001	1.51 ± 1.33	1.95 ± 1.76	0.000
>70	6.25 ± 1.29	6.07 ± 2.17	0.390	5.62 ± 0.91	6.17 ± 1.20	<0.001	1.59 ± 1.78	1.80 ± 1.43	0.629
**GENDER**									
Men	5.70 ± 1.13	5.77 ± 2.07	0.469	5.51 ± 0.82	5.97 ± 1.26	<0.001	1.46 ± 1.75	1.39 ± 0.94	0.609
Women	5.92 ± 0.94	5.47 ± 1.48	<0.001	5.26 ± 0.76	5.78 ± 0.97	<0.001	1.71 ± 1.55	1.67 ± 1.34	0.608

*FPG, fasting plasma glucose; HOMAIR, homeostasis model assessment of insulin resistance*.

### Comparison of lipid metabolism and non-alcoholic fatty liver disease (NAFLD)

Compared to SY, LDL, and TG in NJ were significantly elevated (*P* < 0.001 and *P* = 0.004). HDL was significantly decreased in NJ (1.48 ± 0.31 vs. 1.31 ± 0.32, *P* < 0.001). This trend existed in all age and sex subgroups. TC was significantly elevated in NJ only in females—this difference did not exist in males (Table 2).

**Table 2:** Comparison of blood lipids in these two regions.

	**LDL**			**TG**			**HDL**			**TC**		
	**SY**	**NJ**	**P**	**SY**	**NJ**	**P**	**SY**	**NJ**	**P**	**SY**	**NJ**	**P**
Overall	2.67 ± 0.64	3.23 ± 0.80	<0.001	1.53 ± 1.57	1.57 ± 1.07	0.004	1.48 ± 0.31	1.31 ± 0.32	<0.001	5.08 ± 1.01	5.13 ± 1.45	0.231
**AGE (YEARS)**												
<40	2.42 ± 0.54	2.82 ± 0.62	<0.001	1.33 ± 2.16	1.04 ± 0.58	0.060	1.44 ± 0.29	1.36 ± 0.31	0.011	4.66 ± 0.82	4.58 ± 1.09	0.442
40–50	2.59 ± 0.61	3.17 ± 0.69	<0.001	1.51 ± 1.85	1.50 ± 1.34	0.915	1.46 ± 0.28	1.39 ± 0.32	0.010	4.95 ± 0.95	5.01 ± 1.00	0.452
50–60	2.85 ± 0.72	3.38 ± 0.82	<0.001	1.65 ± 1.45	1.64 ± 1.08	0.920	1.51 ± 0.34	1.33 ± 0.33	<0.001	5.39 ± 1.18	5.40 ± 1.82	0.863
60–70	2.70 ± 0.61	3.30 ± 0.87	<0.001	1.53 ± 1.14	1.77 ± 1.08	0.220	1.50 ± 0.31	1.28 ± 0.32	<0.001	5.14 ± 0.92	5.23 ± 1.53	0.323
>70	2.65 ± 0.60	3.23 ± 0.79	<0.001	1.58 ± 1.10	1.55 ± 0.72	0.302	1.47 ± 0.32	1.23 ± 0.32	<0.001	5.06 ± 0.96	5.07 ± 1.01	0.923
**SEX**												
Men	2.71 ± 0.68	3.16 ± 0.73	<0.001	1.76 ± 2.06	1.82 ± 1.34	0.031	1.46 ± 0.34	1.20 ± 0.30	<0.001	5.16 ± 1.09	5.11 ± 1.66	0.643
Women	2.63 ± 0.60	3.25 ± 0.82	<0.001	1.35 ± 1.01	1.49 ± 0.95	0.000	1.50 ± 0.29	1.36 ± 0.32	<0.001	5.02 ± 0.95	5.14 ± 1.37	0.027

*LDL, low-density lipoprotein; TG, triglyceride; HDL, high-density lipoprotein; TC, total cholesterol*.

**Figure 1:**
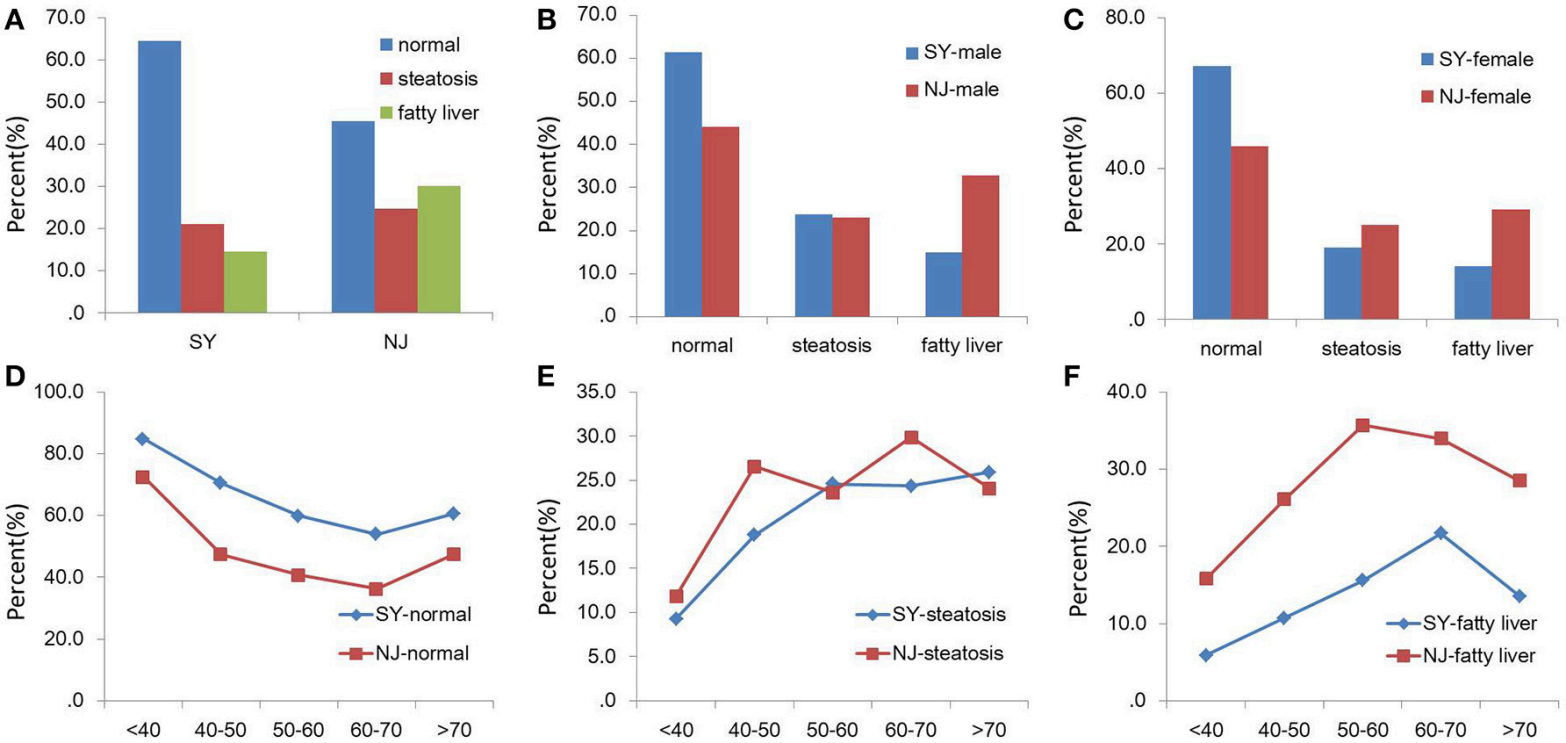
Comparison of NAFLD between SY and NJ. Proportions of normal, steatosis and fatty liver in total **(A)**, males **(B)**, and females **(C)**. Proportions of normal **(D)**, steatosis **(E)**, and fatty liver **(F)** in different age groups in SY and NJ.

There was a significant difference in NAFLD and normal subjects between SY and NJ (*P* = 0.008 and *P* = 0.007). This difference existed both in males and females. As the age increased, the proportion of normal subjects decreased, but the proportion of those with steatosis or NAFLD increased. However, in people over 70 years old, the proportion of fatty liver decreased, whereas the proportion for normal liver increased in both NJ and SY. Additionally, 21.0 and 24.6% subjects had steatosis in SY and NJ, respectively, (*P* = 0.55) (Figure 1).

### Comparison of BMI, NC, WC and HC

There was a significant difference in BMI between these two locations (23.03 ± 3.23 vs. 25.39 ± 3.28, *P* < 0.001). WC and HC in SY were significantly lower than those in NJ (76.82 ± 8.86 vs. 82.77 ± 9.93, *P* < 0.001, 91.27 ± 6.77 vs. 95.55 ± 6.93, *P* < 0.001). Moreover, these differences existed in all age groups and between the sexes. However, NC in SY was lower than in NJ only in males (*P* < 0.001) (Table 3).

**Table 3:** Anthropometric measures between these two places.

	**BMI**			**NC**			**WC**			**HC**		
	**SY**	**NJ**	**P**	**SY**	**NJ**	**P**	**SY**	**NJ**	**P**	**SY**	**NJ**	**P**
Overall	23.03 ± 3.23	25.39 ± 3.28	<0.001	33.60 ± 3.12	33.77 ± 3.80	0.193	76.82 ± 8.86	82.77 ± 9.93	<0.001	91.27 ± 6.77	95.55 ± 6.93	<0.001
**AGE (YEARS)**												
<40	21.95 ± 3.29	23.89 ± 3.43	<0.001	33.06 ± 3.19	33.06 ± 3.63	0.992	72.86 ± 9.08	76.60 ± 12.42	0.001	89.28 ± 6.51	93.12 ± 6.44	<0.001
40–50	23.00 ± 3.04	24.89 ± 3.01	<0.001	33.75 ± 3.11	33.44 ± 3.11	0.243	76.44 ± 8.21	79.22 ± 9.39	<0.001	91.16 ± 5.91	94.55 ± 7.26	<0.001
50–60	23.43 ± 3.10	25.54 ± 2.96	<0.001	33.66 ± 3.10	33.81 ± 3.83	0.542	77.89 ± 8.55	83.08 ± 8.70	<0.001	91.74 ± 6.34	95.51 ± 6.73	<0.001
60–70	23.40 ± 3.24	26.05 ± 3.42	<0.001	33.78 ± 3.00	33.98 ± 3.89	0.417	77.86 ± 8.74	85.30 ± 8.52	<0.001	92.15 ± 7.32	96.57 ± 6.70	<0.001
>70	22.74 ± 3.45	25.77 ± 3.22	<0.001	33.43 ± 3.28	34.35 ± 4.44	0.034	78.08 ± 9.26	87.50 ± 8.79	<0.001	91.01 ± 7.70	97.14 ± 7.02	<0.001
**SEX**												
Men	23.15 ± 3.01	25.63 ± 3.03	<0.001	35.02 ± 2.75	37.11 ± 4.11	<0.001	78.67 ± 8.29	87.14 ± 9.70	<0.001	91.33 ± 6.10	96.19 ± 5.95	< 0.001
Women	22.94 ± 3.38	25.31 ± 3.35	<0.001	32.48 ± 2.92	32.69 ± 2.98	0.130	75.38 ± 9.02	81.35 ± 9.59	<0.001	91.23 ± 7.24	95.35 ± 7.21	<0.001

*BMI, body mass index; NC, neck circumference; WC, waist circumference; HC, hip circumference*.

### Linear regression analysis of metabolic index

HbA1c, FPG, BMI, Log_10_ALT, SBP, LDL, log_10_TG and HDL were used as independent variables. After adjusting for cofactors and covariants, HbA1c BMI LDL and Log_10_TG in NJ were higher than in SY in two models. However, FPG and HDL were relatively lower in NJ. Log_10_ ALT and SBP did not exhibit significant differences in these two places (Table 4).

**Table 4:** Liner regression analysis of metabolic indexs in NJ compared to SY.

	**Model 1**	**Model 2**
**HbA1C**		
NJ (vs. SY)	0.47 (0.40, 0.53)	0.32 (0.25, 0.40)
**FPG**		
NJ (vs. SY)	−0.32 (−0.42,−0.23)	−0.83 (−0.91,−0.76)
**BMI**		
NJ (vs. SY)	2.44 (2.21, 2.68)	1.24 (0.99, 1.49)
**SBP**		
NJ (vs. SY)	2.812 (1.469, 4.155)	−0.106 (−1.657, 1.445)
**LDL**		
NJ (vs. SY)	0.537 (0.485, 0.589)	0.534 (0.481, 0.587)
**Log_10_ TG**		
NJ (vs. SY)	0.025 (0.008, 0.042)	−0.115 (−0.132, −0.098)
**HDL**		
NJ (vs. SY)	−0.183 (−0.206,−0.160)	−0.206 (−0.229, −0.182)

*Model 1, Age, sex, location; Model 2, BMI, HbA1c, SBP, HDL, LDL, TG*.

### Logistic regression analysis of different metabolic status

**Figure 2:**
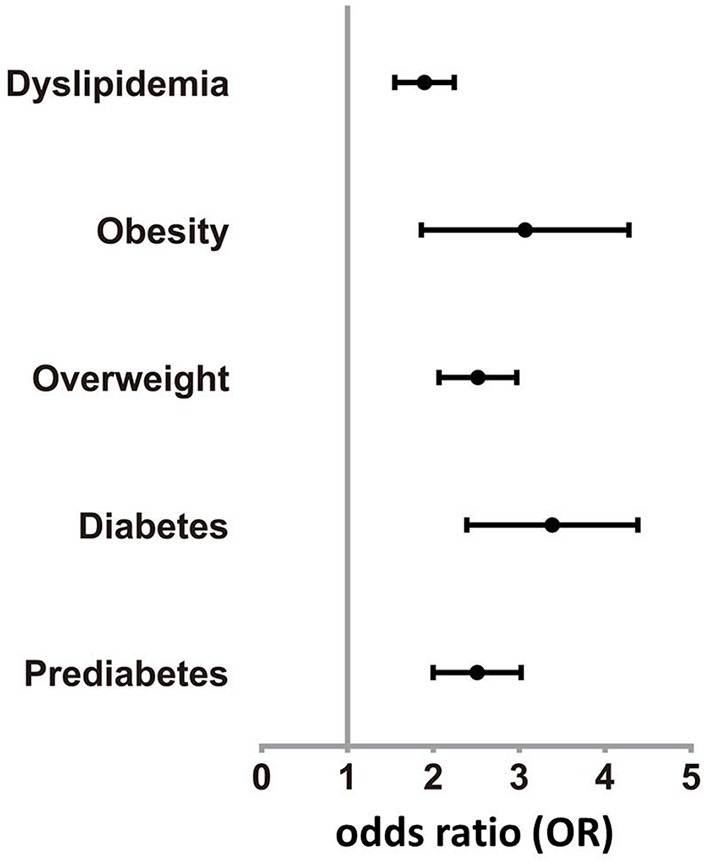
Odds rate of various diseases in NJ. Prevalence of prediabetes, diabetes, overweight, obesity, and dyslipidemia in NJ were higher than SY.

Compared to SY, the odds ratio (OR) of prediabetes and diabetes was 2.475 (95%CI 2.016, 3.038) and 3.287 (95%CI 2.442, 4.425) in NJ (adjusting for exclusion of FPG and HbA1c), respectively. Compared with SY, the OR of overweight and obesity was 2.491 (95%CI 2.079, 2.985) and 2.909 (95%CI 1.947, 4.348) in NJ (adjusting for exclusion of BMI), respectively. Dyslipidemia was 87.5% higher in NJ than in SY (OR = 1.875, 95%CI 1.562, 2.252) (Figure 2).

## Discussion

In the past 30 years and especially in the past 10 years (12), China has experienced the largest scale of urbanization, shifting from agricultural employment to industrial-, commercial- or service-based employment (13). Rapid urbanization is accompanied by unhealthy lifestyles that include a shift in diet from plant food (rich in carbohydrates and fiber) to those with fats, saturated fats and sugars (7) along with a decline in occupational physical activity (PA) (14). For the first time, we compared two places with different urbanization speeds to explore the influence of urbanization on metabolism. There was a significant difference in occupational composition, blood glucose, lipids, NAFLD, and BMI in these two places.

Urbanization changed the occupational composition, where the industry and agriculture (heavy occupational PA) were significantly reduced in NJ. Thus, more people were engaged in the service (light occupational PA). Meanwhile, villagers in NJ lost their farmland, and 36.3% became unemployed or retired early. Finally, residents in NJ had dramatically decreased occupational PA than SY. PA mainly includes occupational PA, commuting PA and leisure time PA (15, 16). Occupational PA constitutes the major source of PA for adults in China (17), while leisure time PA is not popular in China. After occupational alteration, people will lose most of their PA. NJ was previously an agricultural region, and its urbanization will result in occupational PA to be certainly less common (7). Meanwhile, leisure time PA and exercise have not increased as they did in Western countries (13, 17, 18). A previous study indicated that unhealthy lifestyle behaviors such as a lack of PA and high levels of sedentary behavior could significantly increase obesity, type 2 diabetes, and metabolic syndrome (19). Moderate or high levels of occupational PA have been related to reducing the risk of type 2 diabetes (20). In addition, Huang et al. also performed a large scale investigation and found that occupational PA could be important for the prevention of metabolic syndrome in Taiwan (21).

In our study, the prevalence of diabetes was 16.5% in NJ, which is higher than that previously reported both in urban (14.3%), and rural (10.3%) areas (22). The subjects in SY had higher FPG than those in NJ. However, HbA1c in SY was lower than that of NJ. Therefore, we speculated that postprandial plasma glucose (PPG) in SY was lower than in NJ. According to glucose status, all subjects were divided into three categories (normal, prediabetes, and diabetes). With respect to FPG and HbA1c, we deduced that PPG in prediabetes was higher in NJ. Even in normal subjects, PPG was also significantly elevated in NJ. The significant difference in occupational composition of these two places might be caused by decreased occupational PA in NJ accompanied with change of lifestyle. Furthermore, less PA induces accumulation of energy in the body and causes diabetes, obesity, NAFLD and metabolic syndrome (13, 23–25).

Increased LDL and TG and decreased HDL were detected in NJ. However, TC in NJ was higher than that of SY in females but not in males. In people over 70 years old, the proportion of fatty liver decreased, and the proportion of normal liver increased in both NJ and SY. However, both LDL and TC reached their peak at 60 years, after which they decreased. Dyslipidemia is closely related to NAFLD (26, 27). This could partly explain the decline of NAFLD at 70 years of age. In the process of urbanization, the older people retained good life habits, which could be related to the metabolic memory effect.

BMI in NJ was greater than that in SY for all age and sex subgroups. The overall NC was higher in NJ than in SY. However, this trend was more obvious in males. The WC and HC in NJ were higher than in SY. Moreover, the residents in NJ had higher HbA1c, LDL and TC, which were all risk factors for cardiovascular diseases (28).

There are several limitations to our study. First, we did not test PPG in this study, although we could estimate it using FPG and HbA1c. Second, we did not apply metabolic equivalent of task (MET). Third, passage from rural work activities to urban activities is associated with loss of seasonal rhythms and with sleep changes. Sleeping too little or too much can influence insulin resistance and obesity. However, we did not measure sleep duration in our article (29, 30). Forth, we did not consider other indicators (such as wealth index, socioeconomic status, level of income, or chronic stress) vital for urbanization. Fifth, this is a cross-sectional study, and no conclusions of causality can be drawn. Finally, we also lacked the baseline demographic and biochemical data of these two places. In addition to this limitation, the samples were collected at different times.

In conclusion, China is undergoing profound changes in urbanization today. The prevalence of diabetes, obesity, and NAFLD are increased in rapid urbanized areas. Occupational alterations may play a role in this process. Thus, greater attention should be paid in these areas to reduce the prevalence of metabolic disease. Additionally, social determinants that may predict differences in cardiometabolic risk should be addressed.

## Author contributions

YL and NW designed and supervised this investigation. BH and YiC performed this investigation. QL, CZ, and YinC contributed to the data collection. FX and JC provided technical or material support. All authors read and approved the final manuscript.

### Conflict of interest statement

The authors declare that the research was conducted in the absence of any commercial or financial relationships that could be construed as a potential conflict of interest.

## Supplementary material

Supplement Figure 1 Occupational composition of SY and NJ. **(A)** and **(B)** represent the occupational composition in SY and NJ respectively.

Supplement Table 1 Comparison between different places.

Supplement Table 2 Comparison of blood glucose status in these two places.
