# Supplementary material for: Comparison of the Prevalence of Metabolic Disease Between Two Types of Urbanization in China
Source: Front Endocrinol (Lausanne). 2018 Nov 12;9:665. doi: 10.3389/fendo.2018.00665 (PMC6240687; doi:10.3389/fendo.2018.00665)
Supplement: Supplement Figure 1 — Occupational composition of SY and NJ. (A) and (B) represent the occupational composition in SY and NJ respectively. [file Table_1.DOCX]

**Supplement Table 1**. Comparison between different places.

|  | SY | NJ |
| --- | --- | --- |
| Male (y) | 54.16±13.41 | 56.47±12.84 |
| Female (y) | 53.53±13.57 | 55.16±12.42 |
| SBP (mmHg) | 130.03±20.58 | 133.26±20.80 |
| DBP (mmHg) | 77.70±12.23 | 79.67±12.71 |
| Diabetes (%) | 6.5 | 16.0 |
| Prediabetes (%) | 17.9 | 31.0 |
| Normal (%) | 75.7 | 53.0 |

**Supplement Table 2.** Comparison of blood glucose status in these two places.

|  |  | Diabetes | |  | Pre diabetes | |  | Normal | |  |
| --- | --- | --- | --- | --- | --- | --- | --- | --- | --- | --- |
| Glucose status | Age | SY | NJ | P | SY | NJ | P | SY | NJ | P |
| HbA1c | Overall | 7.30±1.53 | 7.54±1.54 | 0.203 | 5.92±0.21 | 5.94±0.20 | 0.152 | 5.07±0.40 | 5.24±0.31 | <0.001 |
|  | <50 | 7.48±1.33 | 7.66±1.52 | 0.745 | 5.89±0.18 | 5.90±0.20 | 0.749 | 4.98±0.44 | 5.16±0.31 | <0.001 |
|  | 50-60 | 7.28±1.67 | 7.77±1.75 | 0.194 | 5.91±0.22 | 5.95±0.22 | 0.292 | 5.14±0.33 | 5.29±0.27 | <0.001 |
|  | 60-70 | 7.28±1.57 | 7.41±1.45 | 0.691 | 5.94±0.22 | 5.96±0.19 | 0.507 | 5.15±0.34 | 5.34±0.29 | <0.001 |
|  | >70 | 7.24±1.41 | 7.49±1.48 | 0.508 | 5.94±0.18 | 5.97±0.18 | 0.414 | 5.17±0.35 | 5.31±0.32 | 0.015 |
| FPG | Overall | 7.61±2.35 | 8.03±2.84 | 0.199 | 5.94±0.89 | 5.29±0.58 | <0.001 | 5.64±0.67 | 4.94±0.47 | <0.001 |
|  | <50 | 7.48±2.69 | 8.95±3.69 | 0.257 | 5.75±0.95 | 5.24±0.62 | 0.001 | 5.43±0.62 | 4.87±0.41 | <0.001 |
|  | 50-60 | 7.57±2.42 | 8.27±3.07 | 0.250 | 5.93±0.87 | 5.24±0.58 | <0.001 | 5.70±0.64 | 4.97±0.44 | <0.001 |
|  | 60-70 | 7.31±2.31 | 7.63±2.42 | 0.526 | 5.98±0.86 | 5.34±0.60 | <0.001 | 5.89±0.65 | 5.00±0.57 | <0.001 |
|  | >70 | 8.17±2.13 | 8.29±3.03 | 0.871 | 6.10±0.91 | 5.35±0.52 | <0.001 | 5.90±0.73 | 5.04±0.50 | <0.001 |

FPG: fasting plasma glucose
