# Supplementary figures and images for: Comparison of the Prevalence of Metabolic Disease Between Two Types of Urbanization in China
Source: Front Endocrinol (Lausanne). 2018 Nov 12;9:665. doi: 10.3389/fendo.2018.00665 (PMC6240687; doi:10.3389/fendo.2018.00665)

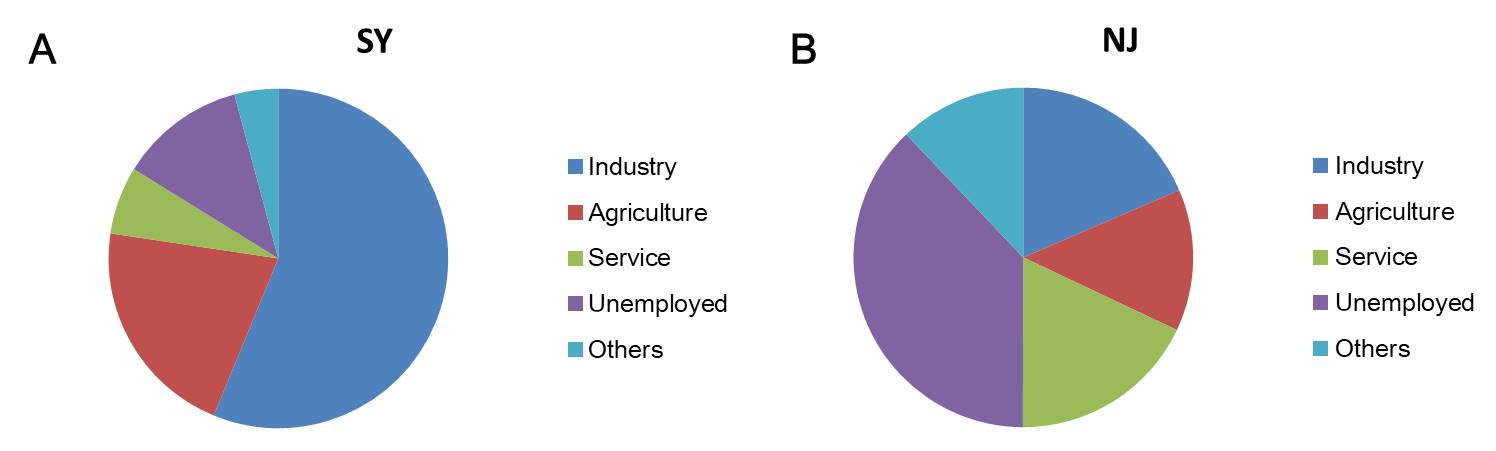

Supplement: Supplement Table 1 — Comparison between different places. [file Image_1.JPEG]
